# Diagnostic Efficacy of Vibration-Controlled Transient Elastography in Patients With Metabolic Dysfunction–Associated Liver Disease and Chronic Hepatitis B

**DOI:** 10.1155/grp/6722810

**Published:** 2024-12-09

**Authors:** Yaoyu Liu, Zhizhen Huang, Xinya Lan, Min Jia, Xiaoting Zheng, Min Hu, Huiying Luo, Luyun Zhang, Xuejing Li, Shaodong Chen, Yunru Li, Huiqing Liang

**Affiliations:** ^1^Hepatology Unit, Xiamen Hospital of Traditional Chinese Medicine, Xiamen, Fujian, China; ^2^Department of Traditional Chinese and Western Medicine, Hunan Provincial Maternal and Child Health Care Hospital, Changsha, Hunan, China; ^3^Fujian Provincial Hospital Rehabilitation Medicine Department, Fuzhou, Fujian, China; ^4^School of Traditional Chinese Medicine, Fujian University of Chinese Medicine, Fuzhou, Fujian, China; ^5^Center for Advanced Kampo Medicine and Clinical Research, Juntendo Graduate School of Medicine, Tokyo, Japan; ^6^Department of Traditional Chinese Medicine, School of Medicine, Xiamen University, Xiamen, Fujian, China; ^7^Liver Diseases Academy of TCM, Beijing University of Chinese Medicine, Beijing, China

**Keywords:** CHB, controlled attenuation parameter, FibroScan, fibrosis, MASLD, steatosis, vibration-controlled transient elastography

## Abstract

**Aim of the Study:** HBV-infected individuals are also presenting with MASLD. However, the value of VCTE for detecting hepatic fibrosis and steatosis in CHB patients concurrent with MASLD is unclear. In patients with combined CHB and MASLD, we intend to assess the diagnostic efficacy of VCTE in determining the extent of fibrosis and steatosis.

**Methods:** This retrospective study involved 368 patients diagnosed with chronic HBV infection combined with MASLD who received liver biopsy and VCTE at Xiamen City Traditional Chinese Medicine Hospital from June 2018 to June 2023. The cutoff values for liver stiffness measurement (LSM) and controlled attenuation parameter (CAP) were determined via the use of the cross-validated area under the receiver operating characteristic (AUROC) curve analyses to identify pairwise fibrosis stage and grade, respectively. The diagnostic statistics were calculated with a 90% fixed sensitivity and 90% specificity.

**Results:** An AUROC of 0.86 (95% CI: 0.76–0.95) was determined by a LSM cutoff value of 11.25 to identify patients with cirrhosis. Patients have the following values: sensitivity, 0.79; specificity, 0.90; PPV, 0.89; and NPV, 0.81. An AUROC of 0.84 (95% CI: 0.76–0.95) was determined by a CAP cutoff value of 313 to identify patients with severe steatotic liver. Patients have the following values: sensitivity, 0.86; specificity, 0.82; PPV, 0.82; and NPV, 0.85.

**Conclusion:**In this investigation of adult patients diagnosed with CHB with MASLD, VCTE demonstrated a robust capability to differentiate cirrhosis and severe steatotic liver.

## 1. Background

Hepatitis B virus (HBV) infections are prevalent throughout the globe, with 5%–6% of individuals in China exhibiting HBsAg positivity [1]. In China, chronic HBV infection has been recognized as the leading causative agent for liver cirrhosis and hepatocellular carcinoma (HCC) prevalence [2]. Metabolic dysfunction–associated liver disease (MASLD) is positively linked with metabolic syndrome and obesity. As rates of these metabolic conditions continue to rise in China, MASLD has emerged at the forefront as the most commonly diagnosed form of severe liver disease and is also responsible for abnormal liver biochemical index measurements during health screenings [3]. There has been recent interest in recent years regarding the rising overlap of HBV infections and MASLD incidence among the general population, raising concerns regarding the health and well-being of the public [4].

A liver biopsy is considered the benchmark technique for assessing the degree of liver fibrosis in MASLD or chronic hepatitis B (CHB) patients. However, this approach has not been widely implemented in clinical settings as it is inherently invasive and associated with the potential for complications, including bleeding and pneumothorax, in addition to being subject to the possibility of diagnostic errors stemming from sample variability [5].

Transient elastography (TE)–based liver stiffness measurement (LSM) techniques can provide a nonsurgical intervention for the detection and evaluation of liver fibrosis. LSM has been widely adopted throughout Europe, China, and the broader Asia–Pacific region owing to its accuracy and ease of implementation [6, 7]. When seeking to detect the progression of fibrosis in CHB patients or to diagnose cirrhosis, LSM is a highly reliable technique [8, 9]. The precision of this approach, however, is markedly compromised in the context of obesity and hepatic steatosis, leading to higher false-positive rates and a greater risk of patient misdiagnosis [10, 11]. The controlled attenuation parameter (CAP) can be acquired during TE examinations at the same time that LSM analyses are conducted, providing a valuable reference for the sensitive identification of fatty liver and an effective tool for the assessment of hepatic steatosis. CAP values in MASLD patients have been reported to be elevated as compared to those in patients without MASLD, and this difference has the potential to affect LSM values, resulting in the misinterpretation or overestimation of the extent of hepatic fibrosis [12]. The diagnostic value of LSM and CAP for assessing liver fibrosis and steatosis severity in individual CHB and metabolic-associated fatty liver has been reported. However, the diagnostic value of LSM and CAP for liver fibrosis and steatosis when both coexist remains unclear. To address these issues, in the present study, a large biopsy sample dataset was leveraged to evaluate the prognostic effect of VCTE in CHB patients comorbid with MASLD. The goal of these analyses was to establish a more precise cutoff value with the potential to serve as a guideline for use in the clinic.

## 2. Methods

### 2.1. Study Subjects

In this retrospective analysis, approximately 368 CHB patients with MASLD were involved who have received liver biopsy and VCTE at Xiamen Traditional Chinese Medicine Hospital from June 2018 through June 2023. VCTE procedures for these patients were performed within 1 week prior to or following liver biopsy. Due to the retrospective design of this investigation, the Institutional Review Board dropped the demand for written consent forms.

### 2.2. Patient Population

Patients eligible for inclusion were (1) ≥ 18 years of age, (2) HBsAg positive for a minimum of 6 months, and (3) individuals with biopsy-confirmed MASLD.

Patients were not included in the study if they exhibited (1) other concurrent liver diseases, including non-HBV viral hepatitis, primary biliary cholangitis, metabolic or hereditary liver diseases, drug-induced liver disease, or autoimmune liver diseases; (2) a history of alcohol use (≥ 40 or ≥ 20 g/day for males and females, respectively); (3) any laboratory or clinical findings consistent with decompensated liver disease; (4) some conditions which can preclude accurate TE measurements, such as the level of alanine aminotransferase (ALT) ≥ 5 times the upper limit of normal (ULN) and total bilirubin (TBIL) levels ≥ 3 times the ULN; or (5) missing laboratory or clinical data.

### 2.3. Liver Biopsy and Evaluation of Curative Efficacy

The implementation of biopsy is based on predicting the efficacy of pegylated interferon therapy with informed consent for the procedure obtained from each patient. All liver samples were evaluated by a trained liver pathologist. Hepatic steatosis was assigned a grade of 0 (< 5% steatosis), 1 (5%–33%), 2 (34%–66%), or 3 (≥ 67%). Using the Scheuer scoring system, fibrosis was categorized into Stages S0–S4, with ≥ S2 being indicative of significant fibrosis.

### 2.4. VCTE

All VCTE procedures were performed by experienced physicians who had independently performed > 10,000 procedures using a FibroScan 502 instrument (EchoSense, France). LSM results were reported in kilopascals, and CAP results were reported in decibels per meter. LSM results were considered reliable and included in this study if they (1) had ≥ 10 valid detections at a specific testing point, (2) exhibited a success rate ≥ 60%, and (3) had an IQR/*M* value < 0.3. CAP values corresponding to reliable LSM values were also deemed reliable and included in this study.

### 2.5. Statistical Analysis

The statistical summary of the study data comprised measures such as percentages, means, and standard deviations. Specificity, sensitivity, positive and negative likelihood ratios (PLR and NLR), and cross-validation of results via area under the receiver operating characteristic (AUROC) curve and 95% confidence intervals (CIs) were among the diagnostic statistics that were examined. The optimal sensitivity and specificity values, as determined by Youden's index, a fixed sensitivity level of 90%, and a fixed specificity level of 90% were utilized in estimating prognostic statistics and LSM cutoffs for elevating pairwise comparisons of different stages of fibrotic severity and CAP cutoffs for elevating pairwise comparisons of various grades of steatosis. By using logistic regression analyses, those variables that were substantially linked to fibrosis (S2) were identified. The data were examined via SPSS 25.0, with a threshold value of *p* ≤ 0.05.

## 3. Results

### 3.1. Study Participants

Approximately 368 patients were eligible to participate in the current study. The mean BMI of these patients was 25.64 ± 3.22 kg/m^2^, and their age was 39.61 ± 9.35 years. HBV DNA was 4.70 ± 2.69 lg IU/mL (Table 1). Of these patients, 4.62%, 69.02%, 13.32%, 5.43%, and 7.61% exhibited Stage 0, 1, 2, 3, and 4 fibrosis based on biopsy results (Table 1). The corresponding distributions of patients with biopsy-based Grade 0, 1, 2, and 3 steatosis were 13.04%, 66.85%, 16.30%, and 3.80%, respectively (Table 1).

### 3.2. The Diagnostic Performance of LSM Values

**Figure 1:**
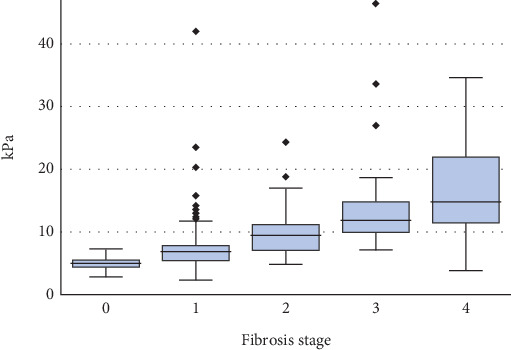
Liver stiffness measurement according to the fibrosis stage.

The respective median LSM scores for all stages (0–4) of fibrosis were 4.9 (4.6, 5.6), 6.8 (5.4, 7.8), 9.4 (7.1, 11.2), 11.8 (10.0, 14.8), and 14.7 (11.5, 22.0) kPa (Figure 1). The cross-validated AUROC values when differentiating between Stage 0 vs. 1–4, 0–1 vs. 2–4, 0–2 vs. 3–4, and 0–3 vs. 4 fibroses were 0.85 (0.78, 0.91), 0.85 (0.80, 0.89), 0.89 (0.83, 0.95), and 0.86 (0.76, 0.95) (Table 2).

When using a fixed sensitivity level (90%), the LSM limit range for differentiating between defined stages of fibrosis was 4.75, 6.55, 8.55, and 8.15 kPa. These LSM cutoff values yielded respective positive predictive value (PPV) measures of 0.61, 0.64, 0.80, and 0.74 when differentiating between defined stages of fibrosis, respectively, with corresponding negative predictive value (NPV) measurements of 0.81, 0.83, 0.88, and 0.87 (Table 2).

When using a fixed specificity level (90%), the respective LSM limit range for differentiating between defined stages of fibrosis was 6.05, 9.65, 10.75, and 11.25, with corresponding PPV of 0.85, 0.86, 0.88, and 0.89 and NPV of 0.74, 0.71, 0.77, and 0.81. The selected cutoff values when analyzing both specificity and sensitivity for distinguishing between defined threshold values of fibrosis were 6.15, 8.05, 8.55, and 11.25 kPa, with corresponding PPV of 0.93, 0.79, 0.80, and 0.89 and NPV of 0.75, 0.81, 0.88, and 0.81 (Table 2).

### 3.3. The Diagnostic Performance of CAP Values

**Figure 2:**
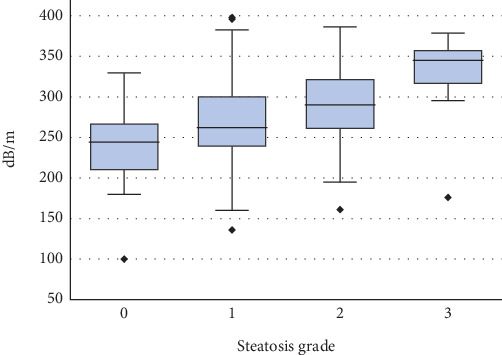
Controlled attenuation parameter according to the steatosis grade.

Median CAP values for all grades (0–3) of steatosis were 244 (210, 267), 262 (239, 300), 290 (261, 321), and 345 (317, 357) dB/m, respectively (Figure 2). The respective cross-validated AUROC values for differentiating between Grade 0 vs. 1–3, 0–1 vs. 2–3, and 0–2 vs. 3 steatoses were 0.70 (0.62, 0.77), 0.71 (0.64, 0.78), and 0.84 (0.71, 0.97). The cutoff values obtained using Youden's index are 275, 271, and 313 between Grade 0 vs. 1–3, 0–1 vs. 2–3, and 0–2 vs. 3 steatoses (Table 3). At a fixed 90% sensitivity level, a threshold limit of 220 dB/m yielded respective specificity, PPV, and NPV of 0.29, 0.56, and 0.74 for the detection of 0–1 grade (≥ 5% steatosis).

At a fixed 90% specificity level, a threshold limit of 284 dB/m yielded respective sensitivity, PPV, and NPV of 0.41, 0.80, and 0.60 for the detection of ≥ 5% steatosis. Respective threshold values after analysis of 0–1 vs. 2–3 and 0–2 vs. 3 grades at a fixed 90% sensitivity level were 241 and 295 dB/m, while the corresponding values at a fixed 90% specificity level were 329 and 334 dB/m. The respective threshold values when analyzing both sensitivity and specificity for differentiating between 0–1 vs. 2–3 and 0–2 vs. 3 grades were 275, 271, and 313 dB/m (Table 3).

### 3.4. Multivariate Logistic Regression Results for Significant Fibrosis

First, we conducted a univariate analysis on age, BMI, HBsAg level, HBeAg level, DNA, PLT, albumin, ALT, AST, GGT, ALP, TBIL, DBil, IBIL, Cho, TG, HDL, LDL, and GLU. Then, we selected statistically significant indicators, age, HBsAg level, HBeAg level, PLT, AST, GGT, TBIL, IBIL, TG, CAP, and E, for multivariate analysis. Multivariate analysis suggested that age (OR = 1.04, *p* = 0.026), GGT (OR = 1.01, *p* = 0.014), TBIL (OR = 0.88, *p* = 0.019), IBIL (OR = 1.24, *p* = 0.003), TG (OR = 0.49, *p* = 0.010), and E (OR = 1.39, *p* = 0.001) are individually correlated with the possibility of significant fibrosis (*p* ≤ 0.05, Table 4).

## 4. Discussion

CHB patients comorbid with MASLD face a greater risk of liver fibrosis, but the ability to effectively and noninvasively identify at-risk patients remains challenging [13]. As VCTE exhibits considerable potential as a nonsurgical intervention for evaluating liver fibrosis and steatosis, no standard measurements have been established for the evaluation of CHB patients comorbid with MASLD. Here, hepatic tissue pathology findings were used as a reference standard to explore the diagnostic efficacy of VCTE measurements with the goal of better informing the clinical application of these measures.

CAP levels have a positive correlation with the chronicity of liver steatosis, and a cross-validated AUROC of 70% was estimated for the classification of patients with 5% or more hepatic steatosis based on histological analysis. A CAP value313dB/m exhibited a NPV of 85% for Grade 0–2 steatosis, thus excluding the potential for Grade 3 steatosis, indicating that there may be clinical value to the application of this cutoff threshold. The accuracy of the 275 dB/m cutoff value for distinguishing between patients with suboptimal steatosis, specifically Grade 2 as opposed to Grade3, was in line with prior reports [14]. This cutoff was in line with the proposed cutoff values published previously [15].

A strong correlation exists between the degree of liver fibrosis and the prognosis of both CHB and MASLD patients. However, the early stages of fibrosis and cirrhosis are often asymptomatic and lack corresponding abnormal indicators or physical signs. In the absence of histopathological analyses of the liver tissue, detecting these conditions can be challenging [16]. While VCTE is not a confirmatory assay, it can aid in the identification of those patients for whom further histological examination may be beneficial while minimizing the risk of liver biopsy procedures being performed in those patients with minimal fibrosis.

LSM values are widely used internationally to examine the severity of fibrosis in CHB patients, and there have also been reports assessing LSM-based analyses of such fibrosis in MASLD patients [17]. Little research to date, however, has focused on noninvasive analyses of hepatic fibrosis in those patients with comorbid MASLD and CHB. Li et al. demonstrated that the performance of a noninvasive test intended to exclude advanced fibrosis did not vary significantly irrespective of fatty liver status. FIB-4 and MASLD fibrosis score (NFS) values have been reported to exhibit respective NPV of 0.80 and 0.78 when excluding advanced fibrosis in CHB and fatty liver subjects [18].

A cutoff value > 8.0 kPa was associated with respective PPV and NPV of 77% and 69% for the detection of substantial fibrosis in a previous group study [19]. Here, an LSM value < 8.1 kPa (8.05) resulted in an NPV of 81% for the exclusion of moderate fibrosis. Less invasive procedures can be performed in those patients with an LSM value < 8.6 kPa (8.55), given that this cutoff was sufficient to exclude advanced fibrosis with around 88% certainty. Increased LSM values can provide higher specificity for the identification of individuals for whom additional confirmatory histological evaluation may be beneficial.

The present study included a relatively low proportion of individuals with S3 and S4 stage disease, potentially impacting the accuracy of these data. The results revealed a direct correlation between measures of liver fibrosis based on TE (from mild fibrosis to cirrhosis) and histological findings for patients with CHB and MASLD. VCTE can be used to evaluate hepatic fibrosis and steatosis in a noninvasive, cost-effective, and easy-to-implement manner. However, this approach cannot obviate the need for liver biopsy, instead serving as an adjunct technique in the clinic that can be used for routine examinations and to exclude those patients with a low risk of hepatic fibrosis while identifying individuals with elevated fibrotic risk who may benefit from undergoing liver biopsy. Future studies have the opportunity to expand on these findings with a larger sample size population to guide the further development of novel or improved noninvasive diagnostic techniques for the evaluation of patients with comorbid CHB and MASLD.

It remains uncertain as to whether concurrent CHB and MASLD can increase the risk of hepatic fibrosis. In one retrospective analysis of 1089 patients with CHB, histological findings indicated that the extent of hepatic fibrosis was elevated for those patients who also had NASH [20]. A separate large retrospective group study that enrolled 6786 CHB patients, however, reported reduced rates of cirrhosis in CHB patients with fatty liver [18]. Several factors can influence hepatic fibrosis. In this study, a multiple regression analysis exhibited that age and GGT, TBIL, TG, and E levels were all independently related to hepatic fibrosis. Factors that are strongly linked to the pathogenesis of MASLD, such as TG and GGT levels, can thus significantly affect fibrosis incidence. The correlation between levels of viral and hepatic inflammatory factors and liver fibrosis was not significant in the present study, potentially because those patients with significant inflammation were excluded from these analyses. Additional in-depth research is thus still warranted to evaluate the relationship between CHB and MASLD in greater detail and the association between this relationship and the incidence of hepatic fibrosis or liver cancer.

## Figures and Tables

**Table 1:** Baseline characteristics of the study population.

**Factors**	**M** **e** **a** **n** ± **SD or n (%)**
*n*	368
Age (years)	39.61 ± 9.35
BMI (kg/m^2^)	25.64 ± 3.22
Laboratory	
HBsAg (IU/mL)	14535.01 ± 33587.03
HBeAg (COI)	163.87 ± 288.68
HBVDNA (lg IU/mL)	4.70 ± 2.69
PLT (109/L)	213.62 ± 59.22
A (g/L)	44.68 ± 3.76
ALT (U/L)	66.15 ± 43.81
AST (U/L)	40.99 ± 29.76
GGT (U/L)	49.29 ± 54.57
ALP (U/L)	75.62 ± 20.94
TBIL (mmol/L)	15.76 ± 7.01
DBil (mmol/L)	6.10 ± 3.48
IBIL (*μ*mol/L)	9.74 ± 5.09
Cho (mmol/L)	4.95 ± 1.11
TG (mmol/L)	1.46 ± 0.91
HDL (mmol/L)	1.17 ± 0.30
LDL (mmol/L)	3.12 ± 1.30
GLU (mmol/L)	5.45 ± 2.82
CAP (dB/m)	271.53 ± 49.67
E (kPa)	8.62 ± 5.52
Steatosis—Mean grade	
Grade 0	48 (13.04%)
Grade 1	246 (66.85%)
Grade 2	60 (16.30%)
Grade 3	14 (3.80%)
Fibrosis—Mean grade	
Grade 0	17 (4.62%)
Grade 1	254 (69.02%)
Grade 2	49 (13.32%)
Grade 3	20 (5.43%)
Grade 4	28 (7.61%)

**Table 2:** Performance diagnostics of liver stiffness measurement assessing the liver fibrosis stage.

**Fibrosis stage: Nonevent vs. event**	**Cross-validated AUROC (95% CI)**	**Cutoff criteria**	**Cutoff (kPa)**	**Sensitivity**	**Specificity**	**Positive predictive value**	**Negative predictive value**
0 vs. 1–4	0.85 (0.78, 0.91)	Youden's index	6.15	0.68	0.94	0.93	0.75
		Sensitivity = 90%	4.75	0.90	0.59	0.61	0.81
		Specificity = 90%	6.05	0.70	0.90	0.85	0.74

0–1 vs. 2–4	0.85 (0.80, 0.89)	Youden's index	8.05	0.82	0.78	0.79	0.81
		Sensitivity = 90%	6.55	0.90	0.50	0.64	0.83
		Specificity = 90%	9.65	0.63	0.90	0.86	0.71

0–2 vs. 3–4	0.89 (0.83, 0.95)	Youden's index	8.55	0.90	0.77	0.80	0.88
		Sensitivity = 90%	8.55	0.90	0.77	0.80	0.88
		Specificity = 90%	10.75	0.73	0.90	0.88	0.77

0–3. vs. 4	0.86 (0.76, 0.95)	Youden's index	11.25	0.79	0.90	0.89	0.81
		Sensitivity = 90%	8.15	0.90	0.68	0.74	0.87
		Specificity = 90%	11.25	0.79	0.90	0.89	0.81

**Table 3:** Performance diagnostics of controlled attenuation parameter in assessing the steatosis grade.

**Steatosis grade: Nonevent vs. event**	**Cross-validated AUROC (95% CI)**	**Cutoff criteria**	**Cutoff (dB/m)**	**Sensitivity**	**Specificity**	**Positive predictive value**	**Negative predictive value**
0 vs. 1–3	0.70 (0.62, 0.77)	Youden's index	275	0.48	0.85	0.76	0.62
		Sensitivity = 90%	220	0.9	0.29	0.56	0.74
		Specificity = 90%	284	0.41	0.90	0.80	0.60

0–1 vs. 2–3	0.71 (0.64, 0.78)	Youden's index	271	0.74	0.61	0.65	0.70
		Sensitivity = 90%	241	0.90	0.30	0.56	0.76
		Specificity = 90%	329	0.31	0.90	0.76	0.57

0–2 vs. 3	0.84 (0.71, 0.97)	Youden's index	313	0.86	0.82	0.82	0.85
		Sensitivity = 90%	295	0.90	0.29	0.76	0.91
		Specificity = 90%	334	0.57	0.90	0.85	0.67

**Table 4:** Multifactor regression analysis for significant fibrosis.

**Variables**	**β**	**SE**	**Z**	**aOR (95% CI)**	**p** ** value**
Age	0.04	0.02	2.22	1.04 (1.00–1.08)	0.026
HBsAg	−0.00	0.00	−0.76	1.00 (1.00–1.00)	0.447
HBeAg	−0.00	0.00	−1.24	1.00 (1.00–1.00)	0.215
PLT	0.00	0.00	0.38	1.00 (1.00–1.01)	0.708
AST	0.00	0.00	0.66	1.00 (0.99–1.01)	0.512
GGT	0.01	0.00	2.46	1.01 (1.00–1.02)	0.014
TBIL	−0.12	0.05	−2.34	0.88 (0.80–0.98)	0.019
IBIL	0.21	0.07	2.92	1.24 (1.07–1.43)	0.003
TG	−0.71	0.28	−2.56	0.49 (0.28–0.85)	0.010
E	0.33	0.06	5.89	1.39 (1.25–1.55)	< 0.001

## Data Availability

The data underlying the results presented in the study are available within the manuscript.

## Nomenclature

AUROC: area under the receiver operating characteristic
CAP: controlled attenuation parameter
CHB: chronic hepatitis B
GGT: gamma-glutamyl transferase
HBsAg: HBV surface antigens
HBV: hepatitis B virus
HCC: hepatocellular carcinoma
LSM: liver stiffness measurement
MASLD: metabolic dysfunction–associated steatotic liver disease
NPV: negative predictive value
PPV: positive predictive value
TBIL: total bilirubin
TE: transient elastography
TG: triglyceride
ULN: upper limit of normal
VCTE: vibration-controlled transient elastography
